# An online international comparison of thresholds for triggering a negative response to the “Surprise Question”: a study protocol

**DOI:** 10.1186/s12904-019-0413-x

**Published:** 2019-04-09

**Authors:** Nicola White, Linda Oostendorp, Victoria Vickerstaff, Christina Gerlach, Yvonne Engels, Maud Maessen, Christopher Tomlinson, Johan Wens, Bert Leysen, Guido Biasco, Sofia Zambrano, Steffen Eychmüller, Christina Avgerinou, Rabih Chattat, Giovanni Ottoboni, Carel Veldhoven, Patrick Stone

**Affiliations:** 1 University College London, London, UK; 2 University of Mainz, Mainz, Germany; 3 Radboud University Medical Centre, Nijmegen, The Netherlands; 4 University Hospital of Bern, Bern, Switzerland; 5 Imperial College London, London, UK; 6 University of Antwerp, Antwerp, Belgium; 7 University of Bologna & Academy of the Sciences of Palliative Medicine, Bologna, Italy; 8 University of Bologna, Bologna, Italy

**Keywords:** Prognosis, Palliative care, Surprise question, Survival, Death

## Abstract

**Background:**

The Surprise Question (SQ) “would I be surprised if this patient were to die in the next 12 months?” has been suggested to help clinicians, and especially General Practitioners (GPs), identify people who might benefit from palliative care. The prognostic accuracy of this approach is unclear and little is known about how GPs use this tool in practice. Are GPs consistent, individually and as a group? Are there international differences in the use of the tool? Does including the alternative Surprise Question (“Would I be surprised if the patient were still *alive* after 12 months?”) alter the response? What is the impact on the treatment plan in response to the SQ? This study aims to address these questions.

**Methods:**

An online study will be completed by 600 (100 per country) registered GPs. They will be asked to review 20 hypothetical patient vignettes. For each vignette they will be asked to provide a response to the following four questions: (1) the SQ [Yes/No]; (2) the alternative SQ [Yes/No]; (3) the percentage probability of dying [0% no chance – 100% certain death]; and (4) the proposed treatment plan [multiple choice]. A “surprise threshold” for each participant will be calculated by comparing the responses to the SQ with the probability estimates of death. We will use linear regression to explore any differences in thresholds between countries and other clinician-related factors, such as years of experience. We will describe the actions taken by the clinicians and explore the differences between groups. We will also investigate the relationship between the alternative SQ and the other responses. Participants will receive a certificate of completion and the option to receive feedback on their performance.

**Discussion:**

This study explores the extent to which the SQ is consistently used at an individual, group, and national level. The findings of this study will help to understand the clinical value of using the SQ in routine practice.

**Trial registration:**

Clinicaltrials.gov NCT03697213 (05/10/2018). Prospectively registered.

**Electronic supplementary material:**

The online version of this article (10.1186/s12904-019-0413-x) contains supplementary material, which is available to authorized users.

## Background

Predicting how long a patient has left to live is an inaccurate and complicated clinical skill [1–5]. Yet it is a clinical skill expected of all medical doctors [6]. In the UK, early identification of people approaching the last year of life can facilitate referral to specialist services and funding, which in turn improves the patient’s quality of life [7–12]. The task of early identification of these patients often falls on General Practitioners (GPs) in the community. The timely identification of patients in the last 12 months of life has been described by GPs as one of the main challenges they face [13]. It has been reported that GPs often wait until very close to death before discussing end of life issues [14] which can often prevent the individual experiencing a “good death” [15, 16]. The “Surprise Question” (SQ) is one such tool available to identify those who might benefit from palliative care. The SQ describes the process whereby clinicians are encouraged to ask themselves whether or not they would “be surprised” if a patient were to die within a specific time (e.g. 12 months, 6 months, 1 month, 7 days). It is suggested that if a clinician “would not be surprised” if a patient were to die within, for example, the next 12 months then this should act as a trigger to adopting a more palliative care approach to their care (e.g. placing the patient on an end-of-life communication register or referring them to specialist palliative care services). Although the original aim of the SQ was not simply to predict survival but to identify people with palliative care needs [17, 18], it is often used as a means to identify people who might be in the last 12 months of life. The prognostic capability of the SQ has been reported to be variable [19–21].

One problem with the SQ is that it is not clear to what extent a death needs to be expected before a clinician would be “surprised” if it did or did not occur. It has previously been highlighted by GPs how subjective and difficult the SQ can be in clinical practice [22]. If there are large variations in these “trigger” values, or “threshold levels”, between clinicians then this would have important implications for how responses to the SQ should be interpreted. The variation between individuals’ threshold levels, and the consistency of the responses to the SQ, have not previously been evaluated. It is also not known which other factors may affect trigger values (e.g. nationality, age, gender, palliative care experience, or years of seniority). A better understanding about when clinicians would, or would not, be “surprised” if a patient were to die would help to standardise and calibrate the use of the SQ. Being aware of the threshold probabilities which trigger “surprise” in different groups may also lead to strategies to improve the accuracy and the consistency with which the SQ is used in clinical practice.

Research by Carel Veldhoven and colleagues [23, 24] reported that the use of an additional alternative Surprise Question “would I be surprised if this patient is still alive after 12 months?”, following a negative response (“No”) to the original Surprise Question, improves the predictive value of the question. Incorporating this question, alongside the original SQ could help improve the prognostic capability of the tool in addition to focusing care where it is needed most. Further research is needed to validate this finding.

In addition to this, there is little evidence about the relationship between the SQ response and the course of action decided upon. What evidence is available suggests that the treatment decisions made at the end of life vary considerable depending on clinician and patient factors [25]. Do GPs consistently pursue the same course of action for patients with similar symptoms, or for those with the same SQ response?

### Objectives

The study has the following objectives:

1. To determine how consistent General Practitioners are in their response to the SQ, and what their “threshold” level is in order to provide a negative response (“No, I would not be surprised”) to the Surprise Question
2. To determine the relationship between the original Surprise Question, the alternative Surprise Question (“Would I be surprised if this person were still alive after 12 months”), and the estimated probability of death occurring in the next 12 months.
3. To understand the course of action decided upon in relation to the prognostic estimates provided.

## Methods

### Methods: participants, interventions, and outcomes

This protocol follows the SPIRIT reporting guidelines for research protocols [26]. The checklist can be found in Additional file 1.

### Study design

The Surprise Study is a non-randomised multicentre online study with a single group of GPs who will complete 20 hypothetical patient summaries, or “vignettes”.

### Study setting

The online study will be distributed to GPs in the following participating countries: Belgium, Germany, Italy, The Netherlands, Switzerland, and the United Kingdom.

### Eligibility criteria

A convenience sample of GPs with a variety of years of experience and seniority across six European countries will be approached to participate.

#### Inclusion criteria

- Registered GP in one of the six participating countries
- Able to read and understand the language in which the study is presented to them

#### Exclusion criteria

- Decline to participate

### Procedure

Participants will receive a Participant Information Sheet (PIS) if they receive an email to participate and/or they will be able to download a PIS directly from the study website. Informed consent will be embedded in the study website and will be obtained via four checkboxes before starting the study. The first will be to consent to participate. The second will be to consent to the results being used in future publications, research and educational packages. The third box will be consent to provide an email address in order to enable participants to log out and return, and for the research team to send reminder emails. The fourth consent box is optional and will ask participants if they wish to receive feedback on their performance. The participant will be reminded that they are free to withdraw at any time. On completion, a debrief page will be provided to remind the participant what the results will be used for. The contact details for the study team will also be displayed should they have any concerns or issues they wish to raise.

Once consented, participants will be asked to read through 20 vignettes of patients with advanced diseases but who have not yet been referred to specialist palliative care services.

### Vignette development

**Fig. 1:**
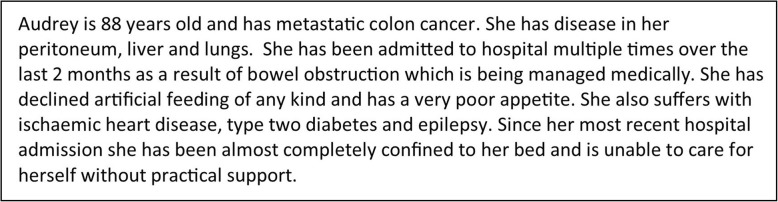
Example hypothetical vignette

The vignettes were constructed by the collaborators in the group and were designed to represent typical patient cases some of whom would be expected to die within 12 months, some of whom would be expected to survive for 12 months and some with an uncertain prognosis. The actual survival of the patients depicted in the vignettes will not be known (since the cases will not represent “real” patients). However the purpose of the exercise is not to evaluate the accuracy of clinicians’ predictions but rather to investigate the level of probability at which respondents would not be surprised if the patient were to die within the next 12 months. The vignettes contain non-specialist information (e.g. they will not contain specialty specific blood test results or diagnostic test results) so that generalist doctors without any specialty-specific training will be able to understand the information provided. See Fig. 1 for an example of a patient vignette.

To ensure an equal distribution of symptoms and disease groups, the vignettes were developed follow a guidance template, informed from the Gold Standards Framework Proactive Indicator Guidance [27], as well as disease specific grading measures (Additional file 2). Each country participating developed a 5 vignettes following this template, from which the final set of vignettes were selected.

### Translation process

We will adhere to the European Organisation for Research and Treatment of Cancer (EORTC) guidelines [28] for the translation process as much as possible within the confines of the resources available for this study. All study documents (the website content, the PIS, the certificate of completion, and the vignettes) will firstly be written in English, verified by the UCL study team. The forward translation into the languages of all countries participating in this study (English, German, Italian, and Dutch) will be completed independently by two translators per language. The translators will compare and form one translated set of documents. The documents will then be back translated to English by a third translator who was not involved in the forward translation. The UCL study team will then review the original version with the back translated version and any differences will be discussed between the translators until the differences have been resolved.

### Outcomes

The primary outcomes will be the continuous estimates of probability of dying within the next 12 months for the SQ (0–100%).

The secondary outcomes will be:

1. Dichotomous response to the SQ (Yes/No)
2. Dichotomous response to the alternative SQ (Yes/No)
3. The options for the course of action selected by the participants in each vignette

### Participant timeline

It is expected that participation should take no longer than 30 min. However, due to the busy work schedules of the participants, they will have the option to “log out” of the study and return at their convenience. This methodology has been used previously and shown to help recruitment as well as minimise attrition [29, 30] Should they not complete the study on the first assessment, they will be sent a reminder email 7 days after they started the study and a final one 3 weeks after starting the study (see Table 1 for the participant timeline). Once recruitment has started, the website will remain open until the end of the study period or until the recruitment target has been met.

**Table 1:** Participant Timeline

	Study Period	Time window (only if not completed at time point 1)		
	Initial access	Reminder email (7 days)^a^	Reminder email (21 days)^a^	Study end
Time point	1	2	3	4
ENROLMENT				
Informed consent	X			
Demographic information	X			
Practice vignette	X			
MAIN STUDY				
20 vignettes	X	X	X	
ASSESSMENTS				
The Surprise Question	X	X	X	
The alternative Surprise Question	X	X	X	
Dichotomous prediction	X	X	X	
Treatment plan	X	X	X	
EXCLUSION (if incomplete)				X

^a^Main study & assessments only need to be completed if not completed at time point 1

### Sample size

Twenty vignettes have been developed. This number of vignettes was estimated in order to keep the task burden to a minimum, to reduce attrition, and to collect enough data points to establish the threshold score for each participant.

We aim to recruit the target sample size of 600 doctors (100 per country) within 8 months of the study opening. This timeframe should be sufficient to allow GPs, with busy working schedules, to complete the study. The sample size is based on calculating the trigger probability to an acceptable level of precision. As there is no current evidence about the trigger probability level for individuals, we assumed a trigger probability of 50%. Using this conservative estimate, aiming at a 4% margin of error (equivalently a precision of 8%), with a level of confidence of 95%, we will need to recruit 600 participants. From previous research that approached a membership body of doctors to participate [31], we anticipate a response rate of approximately 10–15% and therefore will aim to approach 1000–1500 doctors per country.

### Recruitment

The method of recruitment will vary slightly by country in order to accommodate national variations in the required approvals. Participants will be contacted via one of the following methods:

1. An email or receipt of study information within a newsletter from a membership affiliation email
2. An email or word of mouth from a colleague who has participated/heard of the study (snow-balling)

In the UK for example, we could use Local Medical Committees (LMCs), Clinical Commissioning Groups (CCGs) administrators, and the newsletters of the British Medical Association (BMA) and the Royal College of General Practitioners (RCGP) to disseminate a link to the website to participants. In addition to this, we will ask any GP who participates to share the study link with eligible colleagues.

### Methods: data collection, management, and analysis

#### Outcome data collection

Demographic and biographical information about the participants will be collected (location, job title, specialty, setting, age, gender, years of experience since qualifying), and information about their use of the SQ (frequency of use, confidence).

After each vignette, the participant will be asked the following four questions in order to collect data for the primary and secondary outcomes:

1. Would you be surprised if this patient were to die in the next 12 months? (Y/N) (The Surprise Question)
2. Would you be surprised if this patient were to remain alive after 12 months? (Y/N) (The alternative surprise question)
3. What do you think the probability is of this patient dying within the next 12 months? 0% (Certain survival) - 100% (Certain death)
4. What do you think this patient needs? (select more than one if appropriate)

This final question (Q4) will contain a list of potential treatment or care options. The precise wording of these questions will be developed and agreed with the European collaborators to make sure that the questions are culturally and linguistically meaningful.

#### Participant retention

At the completion of the study, participants will be able to download a certificate of participation. Once the study has closed, they will receive feedback on their performance (if they requested this on the consent page). It is hoped that these options, as well as the option to log out of the website and return at a later date, will promote participant recruitment and retention.

### Data management

The database will be designed by a database specialist (CT). The database will be rigorously tested by the UCL study team (NW, LO, VV, & PS) prior to the study starting.

#### Data collection tools

The study is accessed by the participants online and as such, the data will be collected directly from participants.

Each participant will be able to complete a practice vignette prior to completing the main set of vignettes in order to familiarise themselves with the online environment. There will be no time limit for completion so that each participant will be able to take their own time. Participants will not be able to move on to the next page if required information is missing or if responses have been submitted in an incorrect format (e.g. incorrect email format). The UCL study team and the statistician will review the data being collected before, during, and at the start of the recruitment phase to ensure data integrity.

### Statistical methods

A detailed statistical analysis plan will be drawn up in a separate document prior to analysis.

#### Primary outcome analysis

This is an exploratory study to determine how the SQ is utilised by different GPs in different countries. For this reason, we will employ a per-protocol analysis where those participants who do not complete more than 15 vignettes or violate the protocol (e.g. putting the same answer for every vignette) will be removed from the analysis.

We will examine each participant’s responses to the 20 vignettes to calculate the ‘trigger probability’ (or ‘threshold’) for each individual. To calculate this threshold we will examine the participant’s responses to the SQ and the responses to the probability of dying in the next 12 months. We define the threshold as the probability level after which all responses to the SQ are ‘no’. We will also describe the level of uncertainty about this decision. This uncertainty will be calculated by looking at the difference between the lowest probability level with which the participant responded ‘no’ to the SQ and the threshold level.

Three example GP responses are described below to explain this further.

**Fig. 2:**
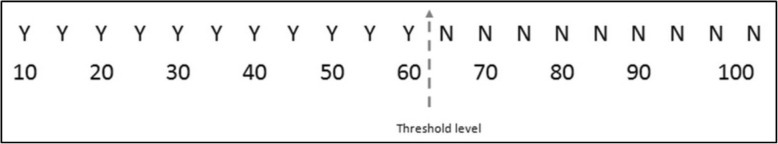
GP 1’s responses

GP 1 responded to 20 vignettes. For each vignette the GP stated the probability of dying and a corresponding answer to the SQ (yes/no). The probability results ranged from 0 to 100%. With each probability the GP also stated ‘yes/no’ to the questions (for ease of display, the individual probability estimates have not been provided but the ‘Y’/‘N’ responses have been placed over the corresponding % to show the point at which the responses changed from Y to N):

In Fig. 2, the probability threshold would be 60%. This GP consistently said ‘no’ to the SQ if the probability of dying was greater than 60%. Consequently the threshold would be 60% and the uncertainty would be 0%.

GP 2 also responded to 20 vignettes.

**Fig. 3:**
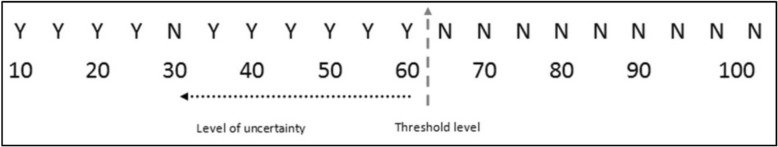
GP 2’s responses

In Fig. 3, the lowest probability level with which the GP responded to ‘no’ on the SQ was 30%. After 60% the GP consistently said ‘no’ on the SQ. Consequently the threshold is 60% but their uncertainty is 30% (= 60–30%).

GP 3 also responded to 20 vignettes.

**Fig. 4:**
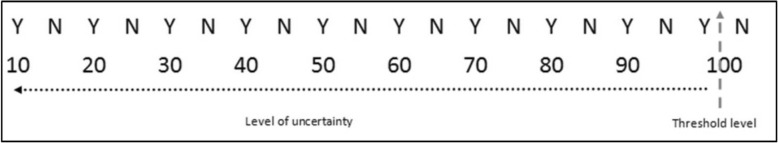
GP 3’s responses

In Fig. 4, the lowest probability for which the GP answered “no” to the SQ was 10%. After 95% the GP consistently said “no”. Consequently the threshold was 95% and the level of uncertainty was 85% (= 95–10%).

Once the thresholds are calculated for all participants, we will use linear regression to explore the differences, if any, in thresholds between countries and other demographic detail collected.

#### Secondary outcome analysis

We will explore the relationship between the alternative SQ and the other responses. We will also describe the actions taken by the clinicians and explore the differences between groups.

## Methods: monitoring

Throughout the study, the research team will review recruitment figures and the study will close once each country has recruited 100 participants. Researchers at UCL will check the responses given by the participants to assess for compliance with the protocol. Participants may be excluded from the analysis if their response record strongly suggests that they did not comply with the study protocol (e.g. all items answered with the same response). The UCL Study Management Group (PS, LO, NW, CT, VV) will be responsible for overseeing the trial and will meet regularly (at least four times per year) to review recruitment figures.

This is a very low risk study. There are no expected side effects of our intervention and this study will not have a Data Monitoring Committee.

### Data management & dissemination

#### Confidentiality

All data will be handled in accordance with the General Data Protection Regulation (GDPR) 2018 [32].

The study will ask the participants for their name and email address as a personal identifier as well as being assigned a participant ID. The purpose of this is to:

1. Enable the participant to log out and back in to the series of vignettes at the same place
2. To contact participants who do not complete the study with a reminder email at the one week and 3 week time points after the last website visit.

The participants will not be asked for any other personal identifiable information. During the study, all data will be kept securely on a web-based database, which is encrypted and password protected. The database will be accessible to approved members of the UCL research team only. Once all data have been reviewed, the names and email addresses will be retained for 12 months after publication, at which point all personal information will be destroyed and only the participant ID will be referenced. This will be explained clearly in the PIS and consent will be sought.

#### Access to the data

In the study, demographic data and outcome data will be collected from participants in accordance with the PIS and this protocol.

The final study database will be downloaded from the website by the research team (Marie Curie Palliative Care Research Department, Division of Psychiatry, UCL) for statistical analysis and UCL will act as the data controller of such data for the study.

The research team (Marie Curie Palliative Care Research Department, Division of Psychiatry, and UCL) will process, store and dispose of the final study database in accordance with all applicable legal and regulatory requirements, including the General Data Protection Regulation (GDPR) and any amendments thereto. Data will be stored electronically, on an encrypted hard drive, with restricted access. The hard drive is maintained by UCL and routinely backed up.

The anonymous dataset will be shared with the other research groups participating in the study.

#### Record keeping and archiving

At the end of the study, all essential documentation will be archived securely by the CI for a minimum of 20 years from the declaration of end of study.

Essential documents are those which enable both the conduct of the study and the quality of the data produced to be evaluated and show whether the site complied with all applicable regulatory requirements.

The sponsor will notify the site when study documentation can be archived. All archived documents will be continued to be available for inspection by appropriate authorities upon request.

#### Dissemination policy

Study results will be published in peer-reviewed, indexed, journals using an open access format, and the results will be presented at national and international conferences. Authorship eligibility will be in accordance with The International Committee of Medical Journal Editors. All proposed publications will adhere to UCL publication policy.

Marie Curie supports this study and we will work with them to disseminate findings through blogs, newsletters and social media.

## Discussion

Four members of the Marie Curie Expert Research Voices Group have reviewed a summary of this study to ensure its aims, objectives, study design and outputs are in line with patient and family needs. All respondents felt it was important to identify patients who may require palliative care early, and they felt the Surprise Question could be a useful way to get doctors to think about the sorts of patients who may or may not require palliative care. Other comments included respondents’ own experiences when a loved one died. These comments were taken in to consideration when developing the study documents.

It is expected that the recruitment of GPs will be challenging due to the nature of their work. We further anticipate that attrition may be an issue due to time constraints on the participants. We have added a function for the doctor to “log out” of the study and return at a more convenient time. We have also endeavoured to minimise the number of vignettes. In addition to this, we will keep the study open as long as possible to try and maximise recruitment. We have adopted recruitment strategies that have proven to be successful in another study which also recruited GPs from the UK [33].

## Additional files

Additional file 1: SPIRIT checklist. (PDF 172 kb)

Additional file 2: Template for vignettes. (PDF 604 kb)

## Abbreviations

BMA: British Medical Association
CCG: Clinical Commissioning Groups
GDPR: General Data Protection Regulation
GP: General Practitioner
LMC: Local Medical Committees
PIS: Participant Information Sheet
RCGP: Royal College of General Practitioners
SQ: Surprise Question
UCL: University College London
UK: United Kingdom
