# Correction to: An online international comparison of thresholds for triggering a negative response to the “Surprise Question”: a study protocol

**DOI:** 10.1186/s12904-020-0518-2

**Published:** 2020-01-27

**Authors:** Nicola White, Linda Oostendorp, Victoria Vickerstaff, Christina Gerlach, Yvonne Engels, Maud Maessen, Christopher Tomlinson, Johan Wens, Bert Leysen, Guido Biasco, Sofia Zambrano, Steffen Eychmüller, Christina Avgerinou, Rabih Chattat, Giovanni Ottoboni, Carel Veldhoven, Patrick Stone

**Affiliations:** 1 University College London, London, UK; 2 University of Mainz, Mainz, Germany; 3 Radboud University Medical Centre, Nijmegen, The Netherlands; 4 University Hospital of Bern, Bern, Switzerland; 5 Imperial College London, London, UK; 6 University of Antwerp, Antwerp, Belgium; 7 University of Bologna & Academy of the Sciences of Palliative Medicine, Bologna, Italy; 8 University of Bologna, Bologna, Italy

**Correction to: BMC Palliat Care**

10.1186/s12904-019-0413-x

Following publication of the original article [1], an error in reference 18 was reported.

Reference 18 did not include all authors. The correct reference should read: Romo RD, Lynn J. The utility and value of the “surprise question” for patients with serious illness. Can Med Assoc J. 2017;189(33):E1072–3.
