# Supplementary material for: An online international comparison of thresholds for triggering a negative response to the “Surprise Question”: a study protocol
Source: BMC Palliat Care. 2019 Apr 9;18:36. doi: 10.1186/s12904-019-0413-x (PMC6461816; doi:10.1186/s12904-019-0413-x)
Supplement: Supplementary file 2 — Template for vignettes. (PDF 604 kb) [file 12904_2019_413_MOESM2_ESM.pdf]

Table 1 The template used for the development of the vignettes

Case	Disease	Gender	Age group	Disease severity	Co-morbidities	# unplanned hospital adms/year	AKPS†	Weight loss in last 6 months	Sentinel Event*	Country to write vignette	
1	Cancer	Male	60-79	mild	none or 1	0-1	70-100	>10%	Yes	Belgium	Germany
2	Cancer	Male	80-100	moderate	multiple	2+	40-60	<10%	No	Switzerland	
3	Cancer	Female	60-79	moderate	none or 1	0-1	40-60	>10%	No	Italy	UK
4	Cancer	Female	80-100	severe	multiple	2+	10 to 30	<10%	No	The Netherlands	
5	CKD	Male	40-60	severe	multiple	0-1	40-60	>10%	Yes	Switzerland	
6	CKD	Male	60-79	mild	none or 1	2+	40-60	<10%	Yes	Germany	Italy
7	CKD	Female	40-60	moderate	multiple	0-1	10 to 30	>10%	No	The Netherlands	Belgium
8	CKD	Female	60-79	moderate	none or 1	2+	70-100	<10%	Yes	UK	
9	Frailty	Male	60-79	moderate	none or 1	0-1	40-60	>10%	Yes	The Netherlands	Switzerland
10	Frailty	Male	80-100	severe	multiple	2+	10 to 30	<10%	Yes	Belgium	
11	Frailty	Female	60-79	mild	none or 1	0-1	70-100	>10%	No	Italy	
12	Frailty	Female	80-100	moderate	multiple	2+	40-60	<10%	No	Germany	UK
13	Heart	Male	60-79	moderate	multiple	0-1	10 to 30	>10%	No	The Netherlands	Belgium
14	Heart	Male	80-100	moderate	none or 1	2+	70-100	<10%	Yes	Germany	
15	Heart	Female	60-79	severe	multiple	0-1	40-60	>10%	Yes	Italy	
16	Heart	Female	80-100	mild	none or 1	2+	40-60	<10%	No	UK	Switzerland
17	Dementia	Male	60-79	mild	none or 1	0-1	70-100	>10%	Yes	The Netherlands	
18	Dementia	Male	80-100	moderate	multiple	2+	40-60	<10%	No	Germany	
19	Dementia	Female	60-79	moderate	none or 1	0-1	40-60	>10%	Yes	Italy	Switzerland
20	Dementia	Female	80-100	severe	multiple	2+	10 to 30	<10%	No	Belgium	UK
21	COPD	Female	40-60	severe	multiple	0-1	40-60	>10%	No	Belgium	
22	COPD	Male	60-79	mild	none or 1	2+	40-60	<10%	Yes	Germany	Italy
23	COPD	Male	40-60	moderate	multiple	0-1	10 to 30	>10%	No	Switzerland	The Netherlands
24	COPD	Female	60-79	moderate	none or 1	2+	70-100	<10%	Yes	UK	

*Sentinel event: bereavement or change of living environment e.g. relocated to nursing home † Australia-modified Karnofsky Performance Scale (AKPS)

Table 2 Disease specific information that was optional for each vignette

Cancer	Deteriorating performance status and functional ability due to metastatic cancer, multi-morbidities or not amenable to treatment – if spending more than 50% of time in bed/lying down, prognosis estimated in months. Persistent symptoms despite optimal palliative oncology. More specific prognostic predictors for cancer are available, e.g. PPS.<br>For disease severity — MILD: Local<br>MODERATE: Regional<br>SEVERE: Distant/Metastatic
Reference	https://www.cancer.gov/about-cancer/diagnosis-staging/staging
Heart Disease	At least two of the indicators below: • Patient for whom the surprise question is applicable. • CHF NYHA Stage 3 or 4 with ongoing symptoms despite optimal HF therapy – shortness of breath at rest on minimal exertion (figure 4). • Repeated admissions with heart failure – 3 admissions in 6 months or a single admission aged over 75 (50% 1yr mortality). • Difficult ongoing physical or psychological symptoms despite optimal tolerated therapy. • Additional features include hyponatraemia<br>For disease severity — MILD: Grade II or better<br>MODERATE: Grade 5-6<br>SEVERE: Grade 7
Reference	http://www.heart.org/HEARTORG/Conditions/HeartFailure/AboutHeartFailure/Classes-of-Heart-Failure_UCM_306328_Article.jsp#.WhbaWVVl-Hs
Chronic Kidney Disease (CKD)	Stage 4 or 5 Chronic Kidney Disease (CKD) whose condition is deteriorating with at least two of the indicators below: • Patient for whom the surprise question is applicable. • Repeated unplanned admissions (more than 3/year). • Patients with poor tolerance of dialysis with change of modality. • Patients choosing the 'no dialysis' option (conservative), dialysis withdrawal or not opting for dialysis if transplant has failed. • Difficult physical or psychological symptoms that have not responded to specific treatments. • Symptomatic Renal Failure in patients who have chosen not to dialyse – nausea and vomiting, anorexia, pruritus, reduced functional status, intractable fluid overload.<br>For disease severity (eGFR)<br>MILD: Stage 3, 30-60 ml/min<br>MODERATE: Stage 4, 15-30 ml/min<br>SEVERE: Stage 5, <15 ml/min
Frailty	• Multiple morbidities. • Deteriorating performance score. • Weakness, weight loss exhaustion. • Slow Walking Speed – takes more than 5 seconds to walk 4 m. • TUGT – time to stand up from chair, walk 3 m, turn and walk back.<br>Disease Severity:<br>There is no specific guidance for frailty however it should be reflected in the AKPS description (Figure 1)
Reference	https://ahsri.uow.edu.au/content/groups/public/@web/@chsd/@pcoc/documents/doc/uow129188.pdf
Dementia	Identification of moderate/severe stage dementia using a validated staging tool e.g., Functional Assessment Staging has utility in identifying the final year of life in dementia. (BGS) Triggers to consider that indicate that someone is entering a later stage are: • Unable to walk without assistance and • Urinary and faecal incontinence, and • No consistently meaningful conversation and • Unable to do Activities of Daily Living (ADL) • Barthel score >3 Plus any of the following: Weight loss, Urinary tract Infection, Severe pressures sores – stage three or four, Recurrent fever, Reduced oral intake, Aspiration pneumonia. NB Advance Care Planning discussions should be started early at diagnosis.<br>For disease severity — **FAST tool (figure 2)**<br>MILD: Grade 1-4<br>MODERATE: Grade 5-6<br>SEVERE: Grade 7
Reference	http://geriatrics.uthscsa.edu/tools/FAST.pdf
COPD	At least two of the indicators below: • Recurrent hospital admissions (at least 3 in last year due to COPD) • MRC grade 4/5 – shortness of breath after 100 metres on level • Disease assessed to be very severe (e.g. FEV1 6 weeks steroids in preceding 6 months, requires palliative medication for breathlessness still smoking.<br>For disease severity — **MRC Dyspnoea scale (Figure 3)**<br>MILD: 0-2<br>MODERATE: 3<br>SEVERE: 4
Reference	https://pcrs-uk.org/mrc-dyspnoea-scale

Additional information on the scales used to describe the specific disease groups whilst writing the vignettes

AKPS ASSESSMENT CRITERIA	SCORE
Normal; no complaints; no evidence of disease	100
Able to carry on normal activity; minor sign of symptoms of disease	90
Normal activity with effort; some signs or symptoms of disease	80
Cares for self; unable to carry on normal activity or to do active work	70
Able to care for most needs; but requires occasional assistance	60
Considerable assistance and frequent medical care required	50
In bed more than 50% of the time	40
Almost completely bedfast	30
Totally bedfast and requiring extensive nursing care by professionals and/or family	20
Comatose or barely rousable	10
Dead	0

Figure 1 The Australia-modified Karnofsky Performance Scale (AKPS)

STAGE	SKILL LEVEL
1.	No difficulties, either subjectively or objectively.
2.	Complains of forgetting location of objects. Subjective word finding difficulties.
3.	Decreased job function evident to co-workers; difficulty in traveling to new locations. Decreased organizational capacity.*
4.	Decreased ability to perform complex tasks (e.g., planning dinner for guests), handling personal finances (forgetting to pay bills), difficulty marketing, etc.
5.	Requires assistance in choosing proper clothing to wear for day, season, occasion.
6a.	Difficulty putting clothing on properly without assistance.
b.	Unable to bathe properly; e.g., difficulty adjusting bath water temperature) occasionally or more frequently over the past weeks.*
c.	Inability to handle mechanics of toileting (e.g., forgets to flush the toilet, does not wipe properly or properly dispose of toilet tissue) occasionally or more frequently over the past weeks.*
d.	Urinary incontinence, occasional or more frequent.
e.	Fecal Incontinence, (occasional or more frequently over the past week).
7a.	Ability to speak limited to approximately a half dozen different words or fewer, in the course of an average day or in the course of an intensive interview.
b.	Speech ability limited to the use of a single intelligible word in an average day or in the course of an interview (the person may repeat the word over and over.
c.	Ambulatory ability lost (cannot walk without personal assistance).
d.	Ability to sit up without assistance lost (e.g., the individual will fall over if there are no lateral rests [arms] on the chair).
e.	Loss of the ability to smile.

Figure 2 Functional Assessment Staging of Alzheimer’s Disease. (FAST)©

MRC Dyspnoea Scale	
**Grade**	**Degree of breathlessness related to activity**
1	Not troubled by breathless except on strenuous exercise
2	Short of breath when hurrying on a level or when walking up a slight hill
3	Walks slower than most people on the level, stops after a mile or so, or stops after 15 minutes walking at own pace
4	Stops for breath after walking 100 yards, or after a few minutes on level ground
5	Too breathless to leave the house, or breathless when dressing/undressing

Figure 3 MRC Dyspnoea Scale

Class	Patient Symptoms
I	No limitation of physical activity. Ordinary physical activity does not cause undue fatigue, palpitation, dyspnea (shortness of breath).
II	Slight limitation of physical activity. Comfortable at rest. Ordinary physical activity results in fatigue, palpitation, dyspnea (shortness of breath).
III	Marked limitation of physical activity. Comfortable at rest. Less than ordinary activity causes fatigue, palpitation, or dyspnea.
IV	Unable to carry on any physical activity without discomfort. Symptoms of heart failure at rest. If any physical activity is undertaken, discomfort increases.

Figure 4 New York Heart Association (NYHA) Functional Classification

The GSF PIG 2016 – Proactive Identification Guidance

Step 1 The Surprise Question

For patients with advanced disease or progressive life limiting conditions, would you be surprised if the patient were to die in the next year, months, weeks, days? The answer to this question should be an intuitive one, pulling together a range of clinical, social and other factors that give a whole picture of deterioration. If you would not be surprised, then what measures might be taken to improve the patient's quality of life now and in preparation for possible further decline?

Step 2 General indicators of decline and increasing needs?

- General physical decline, increasing dependence and need for support.
- Repeated unplanned hospital admissions.
- Advanced disease – unstable, deteriorating, complex symptom burden.
- Presence of significant multi-morbidities.
- Decreasing activity – functional performance status declining (e.g. Barthel score) limited self-care, in bed or chair 50% of day and increasing dependence in most activities of daily living.
- Decreasing response to treatments, decreasing reversibility.
- Patient choice for no further active treatment and focus on quality of life.
- Progressive weight loss (>10%) in past six months.
- Sentinel Event e.g. serious fall, bereavement, transfer to nursing home.
- Serum albumin <25g/l.
- Considered eligible for DS1500 payment.

Step 3 Specific Clinical Indicators related to 3 trajectories

1. Cancer

- Deteriorating performance status and functional ability due to metastatic cancer, multi-morbidities or not amenable to treatment – if spending more than 50% of time in bed/lying down, prognosis estimated in months.
- Persistent symptoms despite optimal palliative oncology. More specific prognostic predictors for cancer are available, e.g. PPS.

2. Organ Failure

Heart Disease

At least two of the indicators below:

- Patient for whom the surprise question is applicable.
- CHF NYHA Stage 3 or 4 with ongoing symptoms despite optimal HF therapy – shortness of breath at rest on minimal exertion.
- Repeated admissions with heart failure – 3 admissions in 6 months or a single admission aged over 75 (50% 1yr mortality).
- Difficult ongoing physical or psychological symptoms despite optimal tolerated therapy.
- Additional features include hyponatraemia <135mmol/l, high BP, declining renal function, anaemia, etc.

Chronic Obstructive Pulmonary Disease (COPD)

At least two of the indicators below:

- Recurrent hospital admissions (at least 3 in last year due to COPD)
- MRC grade 4/5 – shortness of breath after 100 metres on level
- Disease assessed to be very severe (e.g. FEV1 <30% predicted), persistent symptoms despite optimal therapy, too unwell for surgery or pulm rehab.
- Fulfils long term oxygen therapy criteria (PaO2<7.3kPa).
- Required ITU/NIV during hospital admission.
- Other factors e.g., right heart failure, anorexia, cachexia, >6 weeks steroids in preceding 6 months, requires palliative medication for breathlessness still smoking.

Kidney Disease

Stage 4 or 5 Chronic Kidney Disease (CKD) whose condition is deteriorating with at least two of the indicators below:

- Patient for whom the surprise question is applicable.
- Repeated unplanned admissions (more than 3/year).
- Patients with poor tolerance of dialysis with change of modality.
- Patients choosing the 'no dialysis' option (conservative), dialysis withdrawal or not opting for dialysis if transplant has failed.
- Difficult physical or psychological symptoms that have not responded to specific treatments.
- Symptomatic Renal Failure in patients who have chosen not to dialyse – nausea and vomiting, anorexia, pruritus, reduced functional status, intractable fluid overload.

Liver Disease

Hepatocellular carcinoma.

Liver transplant contra indicated.

Advanced cirrhosis with complications including:

Liver Disease *continued*

- Refractory ascites
- Encephalopathy
- Other adverse factors including malnutrition, severe comorbidities, Hepatorenal syndrome
- Bacterial infection current bleeds, raised INR, hyponatraemia, unless they are a candidate for liver transplantation or amenable to treatment of underlying condition.

General Neurological Diseases

- Progressive deterioration in physical and/or cognitive function despite optimal therapy.
- Symptoms which are complex and too difficult to control.
- Swallowing problems (dysphagia) leading to recurrent aspiration pneumonia, sepsis, breathlessness or respiratory failure.
- Speech problems: increasing difficulty in communications and progressive dysphasia.

Parkinson's Disease

- Drug treatment less effective or increasingly complex regime of drug treatments.
- Reduced independence, needs ADL help.
- The condition is less well controlled with increasing "off" periods.
- Dyskinesias, mobility problems and falls.
- Psychiatric signs (depression, anxiety, hallucinations, psychosis).
- Similar pattern to frailty – see below.

Motor Neurone Disease

- Marked rapid decline in physical status.
- First episode of aspirational pneumonia.
- Increased cognitive difficulties.
- Weight Loss.
- Significant complex symptoms and medical complications.
- Low vital capacity (below 70% predicted spirometry), or initiation of NIV.
- Mobility problems and falls.
- Communication difficulties.

Multiple Sclerosis

- Significant complex symptoms and medical complications.
- Dysphagia + poor nutritional status.
- Communication difficulties e.g., Dysarthria + fatigue.
- Cognitive impairment notably the onset of dementia.

3. Frailty, dementia, multi-morbidity

Frailty

For older people with complexity and multiple comorbidities, the surprise question must triangulate with a tier of indicators, e.g. through Comprehensive Geriatric Assessment (CGA).

- Multiple morbidities.
- Deteriorating performance score.
- Weakness, weight loss exhaustion.
- Slow Walking Speed – takes more than 5 seconds to walk 4 m.
- TUGT – time to stand up from chair, walk 3 m, turn and walk back.
- PRISMA – at least 3 of the following:

Aged over 85, Male, Any health problems that limit activity?, Do you need someone to help you on a regular basis?, Do you have health problems that cause require you to stay at home?, In case of need can you count on someone close to you?, Do you regularly use a stick, walker or wheelchair to get about?

Dementia

Identification of moderate/severe stage dementia using a validated staging tool e.g., Functional Assessment Staging has utility in identifying the final year of life in dementia. (BGS) Triggers to consider that indicate that someone is entering a later stage are:

- Unable to walk without assistance and
- Urinary and faecal incontinence, and
- No consistently meaningful conversation and
- Unable to do Activities of Daily Living (ADL)
- Barthel score >3

Plus any of the following: Weight loss, Urinary tract Infection, Severe pressures sores – stage three or four, Recurrent fever, Reduced oral intake, Aspiration pneumonia.

NB Advance Care Planning discussions should be started early at diagnosis.

Stroke

- Use of validated scale such as NIHSS recommended.
- Persistent vegetative, minimal conscious state or dense paralysis.
- Medical complications, or lack of improvement within 3 months of onset.
- Cognitive impairment / Post-stroke dementia.
- Other factors e.g. old age, male, heart disease, stroke sub-type, hyperglycaemia, dementia, renal failure.

Figure 5 The Gold Standard Framework Proactive Indicator Guidance tool
